# How to Test the Purity of Water

**Published:** 1869-03

**Authors:** 


### HOW TO TEST THE PURITY OF WATER.

It is of importance to be able to test the quality of water, not only when for special purposes absolutely pure water is required, but even in cases where such purity is not requisite, it may be of great interest to ascertain of what the impurities consist. The following short notice of the tests for the most commonly occurring impurities will be welcome and useful to many of our readers:—

*Pure Water must satisfy the followinĝ Conditions.*

1. It must have no residue whatever when evaporated in a clear porcelain or platina dish.

2. It must form no precipitate with a solution of nitrate of silver, which would indicate common salt, some other chloride, or hydrochloric acid.

3. It must not precipitate with a solution of chloride of barium, which would indicate a sulphate or sulphuric acid.

4. It must form no precipitate with oxalate of ammonia, as this would indicate some soluble salt of lime.

5. It must not assume any dark or other shade of color when passing sulphuretted hydrogen gas through it, or mixing it with the solution of a sulphide salt, as this would indicate the presence of lead, iron, or some other metal.

6. It must not become milky by the addition of lime-water, or a clear solution of sugar of lead,. as this would indicate carbonic acid.

7. It must not discolor by adding solutions of corrosive sublimate, or chloride of gold, or sulphate of zinc, which discoloring would indicate the presence of organic substances. When boiling water with chloride of gold, the least trace of organic matter will reduce the gold, and color the water brown.

*Results of these Tests.*

1. Almost all spring-waters are found to leave a residue upon evaporation.

2. Common salt is not only found in most springs and rivers, but even in rain-water, many miles inland, when the wind blows from the ocean.

3. Sulphuric acid and sulphates are found in many springs. The Oak Orchard Spring, N. Y., for instance, is very rich in the free acid.

4. Waters from lime regions all contain lime in large quantities; and, in fact, this is the most common impurity of spring-waters.

5. Iron is contained in large quantity in the so-called chalybeate springs; also copper and other metals are encountered; lead incidentally, by the lead tubes through which it often is made to pass.

6. Carbonic acid is the most common impurity; even distilled water is not always free from it. Water will naturally absorb carbonic acid gas from the atmosphere, which latter always contains it; its principal source of supply being derived from the exhalations of man and animals.

7. Organic substances are often found in the water of running brooks, streams, and rivers, and are of course obtained from the vegetation and animal life in the water itself, and from the shores along which it floats.

*Remarks.*

1. The healthfulness of water depends on the nature of the residue left after evaporation; for many chemical and other operations where absolutely pure water is required, the leaving of residue at once proves the water unfit for use.

2. The existence of small quantities of common salt in the water is not objectionable, it being not injurious to health.

3. Sulphuric acid and sulphates may be objectionable for daily use; however, such waters are used medically to stop diarrhœa and excessive tendency to perspiration.

4. Lime-waters do not agree with some constitutions, producing diarrhœa and divers disturbances; very small quantities of lime, however, are not injurious.

5. Iron is healthy, and is a tonic; in fact, this metal and manganese are the only ones which may be used in large doses, not only with impunity, but even with benefit; however, there is also a limit. Overdoses of iron may produce diarrhœa, and slight eruptions of the skin, or pimples.

6. Carbonic acid is not objectionable when drinking the water; on the contrary, it makes it more palatable, and most mineral waters owe their reputation to this substance.

7. Organic substances are perhaps the most objectionable, principally when decaying; such waters may even propagate diseases, and require careful filtering, or boiling, or both, to make them fit for internal consumption.—*Scientific American.* —*New York Medical Journal.*

